# Supplementary material for: Pediatric Coccidioidal Meningitis: A Systematic Review and Proportional Synthesis of Cases Reported in the Fluconazole Era (2000–2025)
Source: J Fungi (Basel). 2025 Oct 1;11(10):713. doi: 10.3390/jof11100713 (PMC12565022; doi:10.3390/jof11100713)
Supplement: Supplementary file 1 [file jof-11-00713-s001.zip › jof-3784695-supplementary.pdf]

Supplementary material:

Table S1. Case reports, case series and retrospective reviews including pediatric patients with CM/CNS involvement, published in the Fluconazole era (2000-2025).

Reference	year	Study type	Center	Total cases (n)	Pediatric cases (n)	Age (range)	Sex (M/F)	Ethnicity	Immunodeficiencies	prior pulmonary disease	CM/CNS involvement	Diagnostic Test	Hydrocephalus	Medical Treatment	Mortality
[34]	2000	Case reports	LAC+USC Medical Center, USA	2	2	3 y	M (n=2)	Hispanic (n=2)	None	1	2	CF, EIA, culture	2	AmB, KET, FLU	0%
[35]	2001	Case reports	Stanford University, California, USA	2	2	10-13y	F (n=2)	NE (live in CA)	LN (n=2)	2	1	culture	NE	AmB, L-AmB, FLU	50% (n=1)
[36]	2004	Case reports	CHRCO, Oakland, CA	2	2	19 mo-16y	M (n=2)	African-american (n=1), Filipino-Latino (n=1)	Asthma (n=1)	1	2	CF	1	FLU, AmB	NE
[37]	2005	Case report	CHLA, Los Angeles, CA	1	1	10 y	M	Mexican	JIA	1	1	CF, biopsy	NE	AmB, FLU, L-AmB, ITRA	0%
[38]	2007	Care report	CHCC, Madera, California, USA	1	1	4 mo	M	NE (live in CA)	No	1	1	CF, EIA, culture	No	FLU	0%
[39]	2008	Case report	CH-HIS, Iowa, USA	1	1	4 y	F	NE	HIES	No	1	culture	Strock	VORI, AmB	0%
[40]	2009	Case Report	UUSOM, Salt Lake City, Utah, USA.	1	1	17 y	F	C	HIES	1	1	EIA, CF, culture	No	AmB, L-AmB, FLU	0%
[41]	2011	Case series	Keck SOM, USC, LA, CA	6	1	16 y	M	Hispanic	No	NE	1	CF	1	FLU, POSA	0%
[42]	2011	Case series	UACOM and SAVAHCS , Tucson,	3	1	6 y	M		No	1	1	Biopsy, culture, CF	NE	AmB	100% (n=1)

			Arizona, USA												
[43]	2012	Case series	Steele CRC, UA, Tucson, USA	6	5	6-16 y	M (n=5).	Hispanic (n=2), White (n=2)	ALL (n=5)	3	1	citology	NE	FLU	0%
[44]	2013	Retrospective review (Jan 26, 1998 – Aug 24, 2011)	SCVMC, San Jose, CA, USA	23	4	5-18 y	M (n=2) F (n=2)	Hispanic (n=2), White (n=2)	HIV (n=3) NE age	NE	4	CF	4	AmB (IT)	0% (1 adult died)
[45]	2013	Retrospective review of samples/case series	NIAID, NIH, Bethesda, MD, USA	5 **	2	9.5-17 y	F (n=2)	Hispanic (n=1), White (n=1)	STAT1 mutation (n=2)	2	2	serology	NE	L-AmB, VORI, POSA. FLU, ITRA, AmB	50% (n=1)
[46]	2013	Retrospective observational study (Jan 1, 2010 – Sep 1, 2011)	CHCC, Madera, California, USA	33	33	6 mo-17 y	M (n=17), F (n=16)	Hispanic (n=21), White (n=7) Asian no-Filipino (n=3), African American (n=2)	No immunocompromised / Any with undiagnosed immunodeficiencies	28	2	CF, culture, biposy	2	L-AmB, IT **AmB-D,** VORI, CASPO, FLU, ITRA, IFN-γ	3% (n=1)
[47]	2013	Retrospective review (Jan 2000 – June, 2012)	UCSF and CHCC	9	9	2 mo-18y	M (n=5) F (n=4)	Asian n=4 (1 Filipino, 2 Laotian, 1 other), White n=4 (2 non-Hispanic, 2 Hispanic/Latino), and Pacific Islander n=1 (Samoan).	Asthma under steroids (n=1)	6	2	CF	2	V/C	No
[3]	2014	Case report	FOM-CU, Egypt	1	1	5 y	M	Sudanese	NE	NE	1	Cytology, biopsy, PCR	1 (edema transependimary in MRI)	No (The patient died prior to receiving antifungal therapy)	100% (n=1)
[22]	2015	Case series + literature review	NIAID, NIH, Bethesda, MD, USA	5***	1	4 y	F	White	STAT3-mutated HIES	1	1	Culture (Bronchoscopy lavage and CSF)	1 (Stroke, hydrocephalus, bilateral VP shunts, and hypercalcemia)	L-AmB, FLU	No

[23]	2016	Retrospective descriptive case series (Jan 1993 – Jun, 2013)	CHLA, Los Angeles, CA	64	64	0-18y	M (n=38), F (n=26)	Hispanic (n=22), No-Hispanic White (n=13) African American (n=11), other (n=13), unknow (n=5)	N=11 (n=9 on immunosuppressants, n=1 HIV+, n=1 asplenia	37	10	Serology (CF), cultures, biopsy/cytology	3	FLU, ITRA, VORI, POSA, AmB (IV or IT)	4.7% (n=3)
[24]	2016	Retrospective case series (Jan 2003 – Dec, 2012)	VCH, Madera, California, USA	13	13	2-11 mo	NE (only one case was M)	Hispanic (n=10), Caucasian (n=2) other, NE (n=1)	NE	13	4	Serology (CF), cultures, biopsy/cytology	NE	FLU, AmB, V/C	0%
[25]	2016	Retrospective analysis of 3 datasets (Jan 1,2000 – Dec 31, 2012)	CDPH, Richmond, CA; VCH, Madera, CA, and Stanford SOM, Stanford, CA, USA	3453	3453 (841† with hospitalizations)	0-17 y	M (n=1839) , F (n=1582)	White (n=471), Hispanic (n=1256), African American (n=84), Asian/Pacific (n=79), Other (n=35)	91 (841†)	568 (841†)	57 (841†)	Confirmed diagnosis in dataset (NE test)	NE	NE	1.3% (11/847 death data)
[26]	2017	Case report	CHOC, Orange, CA, USA	1	1	16 y	M	Chinese American	No	NE	1	CF, culture, cytology	1	FLU	No
[48]	2019	Retrospective case series (Jan 1,2007 – Dec 31, 2016)	VCH, Madera, CA, USA.	78	78	4.5-14.8 y	M (n=43), F (n=35)	Hispanic (n=51), Caucasian (n=13) African American (n=7), Asian no-Filipino (n=6), Asian Filipino (n=1)	N=12 (Immunocompromised n=4; Other comorbidities, n=8)	All cases with extrapulmonary diseases	15	CF, cultures, biopsy/cytology	NE	FLU, L-AmB, VORI, ITRA, V/C	2.6% (2/78)
[32]	2019	Case report	CMC, Long Island, NY. USA	1	1	17 mo.	M	NE	NE	NE	1	CSF culture, genetic habitation, PCR	1	AmB, VORI	No
[49]	2019	Retrospective case series (Jan 1, – Oct 1, 2012)	CHCC, Madera, California, USA	108	108	5 mo-17 y	M (n=56), F (n= 52)	Hispanic (n=75), White(n=19) African American (n=6), Asian /Pacific islander(n=6),	Immunocompromised (n=3)	90	3	Serology (CF), Biopsy, culture	2	AmB, ITRA, FLU, VORI, POSA, V/C	0%

								Other/unknow (n=2)							
[33]	2020	20-year retrospective clinical series (Jan 1998 – Jan 2008)	INNN, Mexico City, Mexico	11	1	19 y	F	Mexican	No	NE	1	Culture, latex agglutination test, pathology, Autopsy, PCR (DNA test)	1	AmB-D, FLU, dexamethasone	No
[50]	2021	Case Report	Dell Med, UT Austin, TX, USA	1	1	16 y	F	NE	JIA	yes	1	CSF serology, CF, culture, Biopsy	NE	FLU, AmB	No
[51]	2021	Case Report	CHCC, Madera, California, USA	1	1	12 (6 y diagnosis with CM)	F	NE	No	NE	1	CF	1	FLU, AmB-D IT, VORI, L-AmB IV, POSA, ISA	No
[52]	2022	Retrospective case series (Jan 1, 2007 – Dec 31, 2020)	CHCC, Madera, California, USA	41 (with Musco skeletal coccidioidomycosis)	41	5.8-14 y	M (n=29), F (n=12)	Latinx (n=27), Caucasian (n=6), African American (n=4), Asian non-Filipino (n=4)	n=12 (Immunocompromised n=3; Other comorbidities, n=9)	24	2	CF, serologies, cultures, Pathology	NE	FLU, ITRA, L-AmB, V/C	2% (n=1)
[53]	2022	Case report+ Literature review	UH-UANL, Monterrey, NL, Mexico.	1	1	7 y	F	Mexican	DAVID syndrome/ c.2611C>T (p.Gln871*)	yes	1	Biopsy, cultures	NE	L-AmB, ITRA	No
[54]	2023	Retrospective case series review (Jan 1, 2000 – Dec 31, 2018)	CHCC, Madera, California, USA	30 (CNS coccidioidomycosis)	30	4.6-15 y	M (n=15), F (n=15)	Latinx (n=14), Caucasian (n=11), African American (n=3), Asian non-Filipino (n=2)	Immunocompromised (n=2)	12	n=30 (25 with CM on MRI)	CF serology, cultures, Biopsy	n=20 (25 with ventriculomegaly in MRI)	FLU, L-AmB, VORI, V/C, AmB-D, ITRA, IFN-γ, steroids, dupilumab	3% (n=1)
[16,29]	2024	Prospective case series (2015-2020)	UMAE No. 71, IMSS. Torreón Coahuila, Mexico.	10	10	6 to 228 mo	M (n=4), F (n=6)	Mexican (n=10)	Elevated IgG (n=2), elevated IgM (n=1), elevated IgE (n=1)	No	10	EIA, CF, biopsy/cytology, PCR	10	FLU, CASPO,	20% (2/10)
[55]	2025	Case series (n=4) + Literature review (n=2)	UCLA, Los Angeles, CA. USA	4	4	10 mo-17 y	M (n=2), F (n=2)	Black (n=1), Mixed-race (n=1), Hispanic (n=1), with-Hispanic (n=1)	Elevated IgE (n=1)	2	4	CF serum and CSF, CSF culture+, Biopsy+	4	FLU, L-AmB, VORI, AmB IT, steroids	50% (2/4)

CDPH = California Department of Public Health, Richmond, CA, USA; CH-HIS = Children's Hospital, Iowa Health Systems, Des Moines, IA, USA; CHCC / VCH = Children's Hospital Central California / Valley Children's Healthcare, Madera, CA, USA; CHLA = Children's Hospital Los Angeles, Los Angeles, CA, USA; CHOC = Children's Hospital of Orange County, Orange, CA, USA; CHRCO= Children's Hospital and Research Center at Oakland, Oakland, CA; CMC = Cohen Medical Center, Long Island, NY, USA; Dell Med, UT Austin = Dell Medical School, The University of Texas at Austin, Austin, TX, USA; FOM-CU = Faculty of Medicine, Cairo University, Cairo, Egypt; INNN = National Institute of Neurology and Neurosurgery, Mexico City, Mexico; JHUSOM = Johns Hopkins University School of Medicine, Baltimore, MD, USA; Keck SOM USC = Keck School of Medicine, University of Southern California, Los Angeles, CA, USA; LAC+USC = Los Angeles County + University of Southern California Medical Center; NIH = National Institutes of Health, Bethesda, MD, USA; NIAID = National Institute of Allergy and Infectious Diseases, Bethesda, MD, USA; SAVAHCS = Southern Arizona VA Healthcare System, Tucson, AZ, USA; SCVMC = Santa Clara Valley Medical Center, San Jose, CA, USA; Stanford SOM = Stanford University School of Medicine, Stanford, CA, USA; Steele CRS = Steele Children's Research Center, University of Arizona, Tucson, AZ, USA; UACOM = University of Arizona College of Medicine, Tucson, AZ, USA; UH-UANL = University Hospital "Dr. José Eleuterio González", Universidad Autónoma de Nuevo León, Monterrey, Mexico; UCSF = University of California, San Francisco Benioff Children's Hospital, San Francisco, CA, USA; UUSOM = University of Utah School of Medicine, Salt Lake City, UT, USA. ALL = Acute Lymphoblastic Leukemia; AmB = Amphotericin B; AmB-D = Amphotericin B deoxycholate; CASPO = Caspofungin; CF = Complement Fixation; CM = Coccidioidal Meningitis; CT = Computed Tomography; CXR = Chest Radiography; EIA = Enzyme Immunoassay; FLU = Fluconazole; GMS = Gomori's Methenamine Silver; HE = Hematoxylin and Eosin; HIES = Hyperimmunoglobulin E Syndrome (Job's Syndrome); IFN-γ = Interferon Gamma; ISA = Isavuconazole; ITRA = Itraconazole; JIA = Juvenile Idiopathic Arthritis (Polyarticular); KET = Ketoconazole; L-AmB = Liposomal Amphotericin B; LN = Lupus Nephritis; PAS = Periodic Acid–Schiff; POSA = Posaconazole; VORI = Voriconazole, V/C= Voriconazole plus Caspofungin; y= year; mo= months. F=female, M=Male. *Case 3 was excluded due to neonatal age (3 weeks old). **n = 2 cases with coccidioidomycosis; n = 3 with confirmed histoplasmosis. ***n = 3 histoplasmosis cases, n = 1 cryptococcosis, and n = 1 coccidioidomycosis.

Table S2. Pediatric individual cases with CM/CNS involvement published in the Fluconazole era (2000–2025).

Reference	Case	Age	Sex	Ethnicity	Immunodeficiency	Pulmonary Involvement	Time to Diagnosis	CM/CNS involvement	hydrocephalus	Other MRI findings	Initial Tratment	Maintenance Tratment	Relapse	FU	Outcome
[34]	1	3y	M	Hispanic	None (HIV-)	Abnormal CXR	7 mo	CSF culture, CSF CF+	yes	NE	AmB	KETO, FLU*	1	9 yr	Alive
	2	3y	M	Hispanic	None (HIV-)	Normal CXR	1 mo	CSF CF, EIA	Yes	NE	FLU	FLU*	1	2 yr	alive
[35]	1	13 y	F	NE	DPGN	CXR abnormal	15 d	BAL cytology +, Autopsy.	No	Disseminated infection	AmB IV + FLU	L-AmB,	0	27 d	Died.
[36]	1	16 y	M	African-American	None	Normal CXR	5 week	CSF CF 1:4, serum CF 1:8. CSF culture neg	No	CT scan: focal area of decreased attenuation in the region of the left posterior thalamus.	FLU +AmB	NE	Initial misdiagnosis meningitis for amoebas	NE (loss of follow-up)	Alive.
	2	19 mo	M	Filipino-Latino	Asthma	CXR mild bronchial thickening	6 days	CSF CF 1:16, serum CF 1:64. CSF culture neg	yes	CT scan: hydrocephalus. MRI: leptomeningeal enhancement surrounding the brainstem into the cervical cord, moderate communicating hydrocephalus and mild periventricular edema.	FLU (12 mg/Kg/d)	NE	Initial misdiagnosis meningitis for amoebas	Yes. (NE time)	Alive.
[37]	1	10 y	M	Mexican	JIA	Miliary CXR pattern	1 mo	CF+, histology+, culture+ (cardiac mass)	NE	NE	AmB IV	FLU, L-AmB*	1	8 mo	Relapse*
[38]	1	4 mo	M	White	None	Abnormal CXR/CT	23 d	Bronchial culture+, CF+, IgG+	No	Cerebellar abscess	FLU	FLU	1	5 mo	Recovered
[39]	1	4 y	F	NE	Job syndrome (HIES)	Normal CXR	4 d	CSF culture +	No	Bilateral basal ganglia and right temporal lobe infarcts	IV VORI	AmB, FLU*	0	NE	Alive/stable
[40]	1	17 y	F	Caucasian	Job syndrome (STAT3 mut)	Abnormal CT	10 d	IgG+, CF+, CSF culture +	No	Focal diffusion restriction (right temporal lobe, posterior internal capsule), no enhancement	IV AmB	FLU*, L-AmB	0	12 d	Recovered

[41]	1	3 y	F	Hispanic	No	NE	NE	CSF CF 1:8, CSF culture +	Yes, VPS*	Ventriculitis	FLU	POSA	1	1 yr	Stable/alive
[42]	1	6 y	M	NE	No	Abnormal CXR	2 mo	Liver biopsy+, CSF culture+, CF 1:2. Autopsy	No	NE	AmB	-		3 d	Died.
[43]	1	16 y	M	Caucasia	ALL	Normal CX	4 weeks	Thumb phalanx KOH, culture +	No	NE	FLU	-	0	20 mo	Alive/stable
[44]	1	14 y	F	White	-	-	--	CSF and serum CF+	Yes	Leptomeningeal enhancement, hydrocephalus +TEF. Intraspinal leptomeningeal enhancement to T10, tonsillar herniation	AmB	-	0	-	alive
	2	8 y	F	Hispanic	-	-	-	CSF and serum CF+	Yes	Severe hydrocephalus, bilateral MCA/ACA infarcts, basal ganglia; angiography: diffuse ICA/MCA/ACA/PCA narrowing	AmB	-	0	-	Alive
	3	18 y	M	Hispanic	-	-	-	CSF and serum CF+	Yes	Moderate hydrocephalus, TEF, MRI and angio normal	AmB	-	0	-	alive
	4	5 y	M	White	-	-	-	CSF and serum CF+	yes	Severe hydrocephalus, infarcts (internal capsule/corona radiata),	AmB	-	0	-	alive

										Angio: MCA/ACA/PCA narrowing					
[45]	1	14 y	F	Hispanic	STAT1 mut c.1057G>A (E353K)	Abnormal CT	3 y	Skin biopsy+	NE	CT: diffuse progression, osteomyelitis (C6–T5), liver and spleen lesions. Intramodular T9 lesion	L-AmB, VORI	POSA	1	2 y	Survived
	2	9.5 y	F	Withe	STAT1 mut c.800C>T (A267V)	Abnormal CXR	3 weeks	Serology+	NE	Ring-enhancing CNS lesions in brain and cerebellum	FLU	ITRA. AmB IO, V/C, POSA, esteroides	1	7.5 yr	Died
[46]	1	3 y	M	NE	None	Normal CXR	16 d	Serologies+ (CF, precipitins), culture or biopsy+	Yes, VP shunt	fatal meningitis	L-AmB, VORI+CASPO	INT-γ,	1	95 d	Died
	2	12 y	M	NE	None	Normal CXR	NE	Serologies (CF, precipitins), culture or biopsy	Yes, VP shunt	Basilar meningitis, focal cerebritis, widespread spinal cord disease	L-Amb, VORI	L-AmB+ FLUCO*, INT-γ, AmB-D IT.	0	241 d	Alive/stable
[47]	1	2 mo	F	Caucasian	NE	Self-limited pneumonia	5 mo	CF >1:256, CSF CF 1:8	Yes, VP shunt	NE	FLU	V/C, FLU*	1	8 y	Alive/stable
	2	18 y	M	Asian/Laotian	No	NE	NE	CF 1:32, CSF CF 1:2	Yes, shunt	Progressive meningitis, cerebritis, myelitis	FLU, VORI, IV L-Amb, IT AmB-D	V*/C	1	7 mo	Progressive disease/neurological deterioration
[3]	1	5 y	M	Sudanese	No	NE. Liver/GI coccidioides	40 d	Liver culture+, PCR -	Yes	MRI with transependimary edema + rounding of third ventricle, occipital infarction	No antifungal therapy started	Not started	1	3 d	Died.
[22]	1	4.5 y	F	White	STAT3 HIES, V713M	Pulmonary disease	NE	BAL+, CSF culture+,	Yes, VP shunt	Hydrocephallus bilateral. Stroke (internal capsule cerebral vascular), hypercalcemia	L-AmB	FLU*	0	7 y	Stable/alive
[26]	1	16 y	M	Chinese/American	No	NE. Skin lesion	CF 1:128, CSF culture +, skin	6 weeks	Yes. VP shunt	MRI supported normal	FLU	FLU*	1	1 y	Alive/stable

							punch biopsy +								
[32]	1	17 mo	M	NE.	No	NE	15 mo	CSF cultur+, PCR+	Yes, VP shunt	Recurrent enlargement of the ventricles	IV AmB + VORI	FLU*	1	4 yr	Alive, controlled
[49]	1	6 yr	M	Caucasian	No	NE	31 d	IgG CF 1:64	No	CNS and bone involvement, meningitis with some spinal disease, dissemination at diagnosis	FLU, AmB, V/C -		1	112 d	Alive/Stable
	2	14 yr	F	Hispanic	Immunocompro mised	NE	145 d	IgG CF 1:2048	yes	meningoencephalitis including brainstem, hydrocephalus, dissemination Identified at Initial Diagnosis	FLU, AmB		0	8 d	Alive/Stable
	3	1 y	M	Hispanic	Nonee	NE	39 d	IgG CF 1:2	yes	Meningitis, obstructive hydrocephalus, spine nodularity, myelitis	FLU, AmB, V/C		0	272 d	Alive/Stable
[33]	1	19 y	F	Mexican	No risk factors	NE	NE	Culture +, serology (LAT), Pathology, PCR (DNA test)	yes	NE	AmB-D, FLU, Dexa		1	2 yr	Alive
[50]	1	16 yr	F	NE	JIA	Abnormal MRI (necrotizing pneumonia)	3 weeks	IgG CF 1:256 CSF CF 1:4. Lung aspirated and CSF culture+. Lung biopsy+	No	Enhancement of the left temporal lobe, infarct temporal, thickened and infiltrated infundibulum. Choroidal lesions	FLU, AmB	FLU*	0	6 mo	Alive/Stable
[51]	1	6 y	F	NE	None	NE	1	CF 1:4, CSF ID+	Yes, VP shunt	Enhancement of the r prepontine cisterns and lumbosacral region , temporal lesion , hemorrhage in prepontine cistern and IFV	FLU	VORI, IT AmB-D, POSA, ISA*	0	6 yr	Alived/stable
[52]	1	6 yr	NE	African American	Asthma	NE	No	CF 1:64	NE	Skull, pelvis, metatarsal lesions	ITRA	-	1	2 y	Relapse
[53]	1	7 y	F	Mexican	DAVID syndrome (CVID mutation c.2611C>T (p.Gln871*)	Miliary infiltrate in CT	15 mo	Node biopsy+, Subgalea abscess culture+	NE	Brainstem lesion, osteomyelitis, mastoid collection. Subgalea abcess parito-occipital	L-AmB ,	ITRA*, Ig replacement	1	1 yr	Alive/Stable
[16,29]	1	6 mo	F	Mexican	No	NE	90 d	CSF IEA, CF 1:32	Yes, shunt	Asymmetric hydrocephalus, IFV	FLU	FLU*	1	6 mo	Died
	2	19 mo	M	Mexican	No	NE	60 d	CSF EIA , CF 1:2	Yes, shunt	-	FLU	FLU*	0	24 mo	Alive/stable

	3	24 mo	M	Mexican	Hiper-IgG	NE	90 d	CSF EIA, PCR+	Yes, shunt	-	Antituberculosis drugs + FLU	FLU*	1	12 mo	Alive/stable
	4	84 mo	F	Mexican	Hiper-IgG	NE	720 d	CSF cytology	Yes, shunt	Asymmetric hydrocephalus, cerebral vasculitis and IFV	CASPO, FLU	FLU*	1	3 mo	Died
	5	108 mo	M	Mexican	No	NE	90 d	CSF EIA, CF 1:6	Yes, shunt	cerebral vasculitis	Antituberculosis drugs + FLU	FLU*	0	14 mo	Alive/controlled
	6	109 mo	M	Mexican	No	NE	90 d	CSF EIA, CF 1:2	Yes, shunt	Asymmetric hydrocephalus	FLU	FLU*	1	18 mo	Alive/stable
	7	156 mo	F	Mexican	Hiper-IgM	NE	90 d	CSF EIA, CF 1:2	Yes, shunt	Asymmetric hydrocephalus	FLU	FLU*	0	14 mo	Alive/stable
	8	204 mo	F	Mexican	Hyper-IgE	NE	30 d	CSF cytology	Yes, shunt	Asymmetric hydrocephalus	FLU	FLU*	0	12 mo	Alive/stable
	9	228 mo	F	Mexican	No	NE	72 d	CSF EIA, CF 1:2	Yes, shunt	Asymmetric hydrocephalus	FLU	FLU*	1	18 mo	Alive/stable
	10	228 mo	F	Mexican	No	NE	90 d	CSF EIA, CF 1:6	Yes, shunt	Asymmetric hydrocephalus	Rifampicin, FLU	FLU*	1	18 mo	Alive/stable
[55]	1	5 y	F	Black	No	Pulmonary disease	NE	CSF serum 1:128. EVD and CSF culture+	VPS, ventricular Ommaya, cisternal Ommaya	CT scan: Hydrocephallus	FLU+L-AmB Dexamethasone	AmB IT	1	8.5 mo	Deceased—care withdrawn
	2	10 mo	M	Mixed-race	No	pulmonary nodules and mediastinal adenopathy	Diagnosis at 4 mo of age	CSF serum 1:128, after 1:1024. CSF CF 1:8, after 1:512. Culture + in lip lesion	Yes, VPS and Cisternal Omaya	MRI brain: severe basilar meningitis, hydrocephalus and spinal cord disease.	FLU 12 mg/kg/d + AmB L	AmB IT. Lifelong Isavuconazole therapy	1	9 mo	Survived
	3	35 mo	F	Hispanic	No	NE	Age at 26 mo. with shunt for hydrocephalus	CSF serum 1:128. CSF culture+	Yes, VPS and EVD, Rickman	CT scan: enlarged ventricles and bilateral subdural hematomas, due to previous over-shunting of CSF	AmB IV	AmB IT. Lifelong fluconazole therapy	1	4 years (6 weeks after treatment).	Survived
	4	17 y	M	White-Hispanic	Hyper IgE	NE	Diagnosis 1.5-year prior admission	CSF serum 1:512. CSF 1:1. Skin and dural biopsy +. DNA exome sequencing dural biopsy neg	Yes, Frontal ventriculostomy and VPS, cervical Ommaya	Brain MRI: hydrocephalus, nodular leptomeningeal enhancement in the basilar cisterns and scattered parenchymal lesions. Repeat MRI: ten new ring-enhancing foci in the subcortical white matter of the cerebrum and cerebellum	L-AmB +VORI, POSA	AmB IT	1	12 days	Died -acute decompensation

compatible with small coccidioidal abscesses and diffuse nodular leptomeningeal enhancement in the cervical, thoracic, and lumbar spine with complete effacement of the cervical spinal space

ACA: anterior cerebral artery; ALL: acute lymphoblastic leukemia; AmB: amphotericin B; CASPO: Caspofungin; CF: complement fixation; CNS: central nervous system; CSF: cerebrospinal fluid; CT: computed tomography; d: days; DPGN: diffuse proliferative glomerulonephritis (lupus nephritis) treated with cyclophosphamide; CXR=chest radiography, EIA: enzyme immunoassay; FLU: fluconazole; FU: follow-up; HIES: hyper-IgE syndrome; ICA: internal carotid artery; INT-γ: interferon gamma; ITRA: itraconazole; JIA: juvenile idiopathic arthritis; L-AmB: liposomal amphotericin B; LoS: hospital length of stay; MCA: middle cerebral artery; mo: months; MRI: magnetic resonance imaging; ND: not described; NE: not evaluated; PCA: posterior cerebral artery; POSA: posaconazole; VP shunt: ventriculoperitoneal shunt; VORI: voriconazole; V/C= Voriconazole plus Caspofungin, d: days, mo: months, y: years. F: female, M: Male. *Lifelong or long-term azole.

Table S3. Variables in the logistic regression model (Backward: LR) evaluating adverse outcomes (progression, relapse, or death) in pediatric cases with CM (n = 48).

Step	Variable	OR	95% CI Lower	95% CI Upper	p-value
1	Edad > 5 años	1.302	0.271	6.264	0.742
	Immunodeficiency	2.430	0.428	13.798	0.316
	Hydrocephalus	1.408	0.209	9.494	0.725
	Serum CF titers ≥1:16	5.349	1.149	24.900	0.033
	Fluconazole first-line treatment	0.412	0.087	1.956	0.264
	Lifelong azole	0.292	0.056	1.525	0.144
	Constant	0.321	—	—	0.313
2	Immunodeficiency	2.513	0.453	13.955	0.292
	Hydrocephalus	1.330	0.205	8.636	0.765
	Serum CF titers ≥1:16	5.349	1.159	24.678	0.032
	Fluconazole first-line treatment	0.421	0.090	1.975	0.273
	Lifelong azole	0.286	0.055	1.484	0.136
	Constant	0.385	—	—	0.328
3	Immunodeficiency	2.212	0.501	9.759	0.295
	Serum CF titers ≥1:16	5.195	1.147	23.534	0.033
	Fluconazole first-line treatment	0.421	0.091	1.954	0.269
	Lifelong azole	0.318	0.072	1.398	0.129
	Constant	0.471	—	—	0.286
4	Serum CF titers ≥1:16	5.065	1.169	21.950	0.030
	Fluconazole first-line treatment	0.458	0.102	2.051	0.307
	Lifelong azole	0.304	0.069	1.331	0.114
	Constant	0.621	—	—	0.452
5	Serum CF titers ≥1:16	4.500	1.097	18.465	0.037
	Lifelong azole	0.250	0.060	1.040	0.057
	Constant	0.444	—	—	0.139

Footnote:

OR = odds ratio; CI = confidence interval; CF = Complement Fixation. Dependent variable: adverse outcome (progression, relapse, or death). Logistic regression performed using the backward likelihood ratio (Backward: LR) method.
